# Multiple cystic lung disease in an adolescent boy

**DOI:** 10.11604/pamj.2016.23.208.8672

**Published:** 2016-04-20

**Authors:** Ozgur Sogut, Demet Tas

**Affiliations:** 1 Haseki Training and Research Hospital, Department of Emergency Medicine, Istanbul, Turkey

**Keywords:** Multiple cystic lung disease, adolescent, giant lung cyst

## Image in medicine

A 15-year-old previously healthy boy was involved in a traffic accident and presented to our emergency department (ED) with complaints of chest pain and mild shortness of breath. On physical examination, he had complained for the last 2 hours of the right hemithorax pain in the fifth intercostal space radiating to the right midaxillary line. He had no past medical or surgical history. Chest x-ray demonstrated doubtful multiple thin-walled cystic lesions in the right lung (A). Computed tomography (CT) scan of thorax revealed a well- defined thin-walled, and 9.97 × 8.78 cm in diameter giant air cyst occupying the right upper lobe parenchyma with multiple air-space cysts (B). The patient was finally diagnosed as a multiple cystic lung disease and he underwent elective thoracic surgery for cyst removal. Cystic lung disease is defined as intrapulmonary air-containing multiple cysts surrounded by sharply demarcated thin walls. CT scanning of thorax is more sensitive than chest radiography in the detection and the distribution of lung cysts. Patients with cystic lung disease may be asymptomatic or present with nonspecific symptoms, such as chronic cough or shortness of breath. They are at increased risk for spontaneous pneumothorax. Surgical treatment of multiple cystic lung disease plays a crucial role in the prevention of pneumothorax.

**Figure 1:**
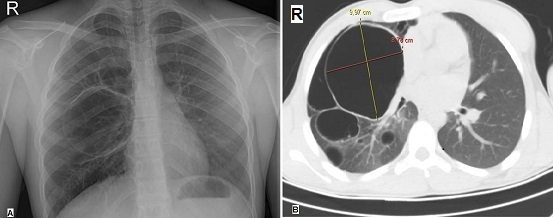
Multiple cystic lung disease. A) chest radiograph shows multiple doubtful cystic lesions in the right lung; B) chest CT scan shows a giant cyst in the right lung with multiple varying sizes and shapes air-filled cysts

